# Dynamic Changes in the Gene Expression Patterns and Lipid Profiles in the Developing and Maturing Meibomian Glands

**DOI:** 10.3390/ijms23147884

**Published:** 2022-07-17

**Authors:** Igor A. Butovich, Amber Wilkerson

**Affiliations:** Department of Ophthalmology, University of Texas Southwestern Medical Center, Dallas, TX 75390-9057, USA; amber.wilkerson@utsouthwestern.edu

**Keywords:** meibomian glands, meibum, meibogenesis, lipidomics, transcriptomics, single-cell RNA sequencing, development, organogenesis, wax ester, cholesteryl ester

## Abstract

Meibomian glands (MGs) and their holocrine secretion—meibum—play crucial roles in the physiology of the eye, providing protection from environmental factors and desiccation, among other functions. Importantly, aging was implicated in the deterioration of the morphology and functions of MGs, and the quantity and quality of meibum they produce, leading to a loss of its protective properties, while the meibum of young individuals and experimental animals provide ample protection to the eye. Currently, the molecular mechanisms of meibum biosynthesis (termed meibogenesis) are not fully understood. To characterize the physiological changes in developing and maturing MGs, we studied the lipidomes and transcriptomes of mouse MGs ranging from newborns to adults. The results revealed a gradual increase in the critical genes of meibogenesis (such as *Elovl3*, *Elovl4*, *Awat2*, and *Soat1*, among others) that positively correlated with the biosynthesis of their respective lipid products. The MG transcriptomes of young and adult mice were also analyzed using single-cell RNA sequencing. These experiments revealed the existence of multiple unique populations of MG cells (meibocytes, epithelial cells, and others) with specific combinations of genes that encode meibogenesis-related proteins, and identified clusters and subclusters of cells that were tentatively classified as meibocytes at different stages of differentiation/maturation, or their progenitor cells. A hypothesis was formulated that these cells may produce different types of lipids, and contribute differentially to the Meibomian lipidome.

## 1. Introduction

Recent advances in transcriptomic and lipidomic analyses of human and rodent Meibomian glands (MGs) [1,2,3,4,5] have laid a foundation for a better understanding of the mechanisms of *meibogenesis* [4,6], which is defined as a network of concerted enzymatic reactions and corresponding regulatory mechanisms that lead to the formation of meibum—a lipid-rich secretion produced by MGs. The concept of meibogenesis has unified various metabolic sub-pathways that were predicted to result in the formation of a very diverse group of, typically, extremely long-chain (ELC) Meibomian lipids, which comprise meibum [6]. This group includes a number of lipid classes, e.g., homologous series of wax esters (WEs) and cholesteryl esters (CEs) as two major components, triacylglycerols (TAGs), free cholesterol (Chl), cholesteryl sulfate, free fatty acids, ceramides, sphingomyelins (SMs), phospholipids (PLs), and a range of other species as minor components. Expectedly, the biosynthetic pathways that produce these diverse groups of lipids are quite convoluted and involve a large group of genes, some of which (such as *Elovl1*, *Elovl3*, *Elovl4*, *Elovl6*, *Soat1*, *Far1*, *Cyp4f39*, and *Awat2*) have been studied and described in earlier publications [7,8,9,10,11,12,13,14,15,16,17]. Notably, the latest publications on the topic by independent teams provided additional support to the original concept of meibogenesis [4,6], as the inactivation of its critical genes led to expected changes in the Meibomian lipidome. There is little doubt that currently ongoing and future studies will provide additional details regarding the mechanisms of meibogenesis and fill out existing gaps in our knowledge of the process. One of those gaps is the dynamic changes in the MG transcriptome and lipidome in developing and maturing MGs. Previously, progression from early adulthood to advanced age was shown to have only minimal impact on the chemical composition of meibum in healthy humans [18], but was linked to a decrease in the rate of meibocyte differentiation [19] and changes in multiple signaling pathways [20]. However, no detailed information on the changes in the lipidome of *developing* MGs (which were reported to become fully matured in mice at P15 or so [21]) and genes of meibogenesis is currently available. Therefore, the goal of this study was to conduct transcriptomic and lipidomic analyses of mouse MGs, starting with Theiler stage P0 to about P80, and identify the major sub-pathways of meibogenesis that change with MG maturation.

## 2. Results

### 2.1. Lipidomic Analyses of Developing and Maturing MGs

#### 2.1.1. Unbiased, Untargeted Analyses of MG Lipids

**Figure 1:**
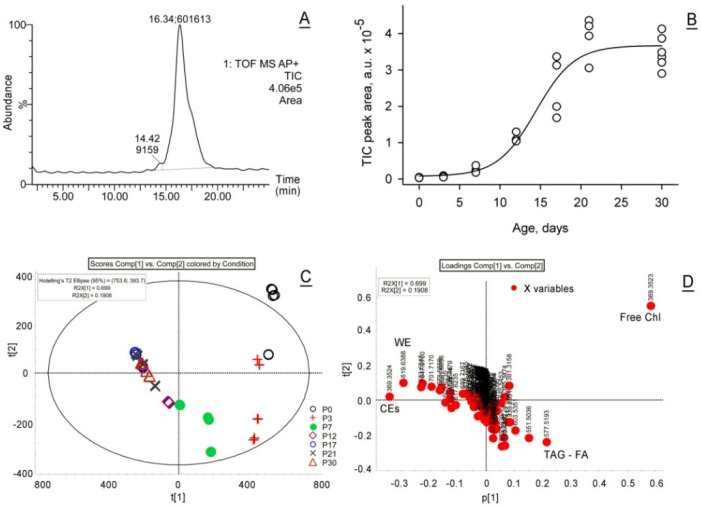
Lipidomic LC–MS analyses of mouse Meibomian lipids revealed the gradual accumulation of meibum in the tarsal plates and its qualitative changes in developing and maturing meibomian glands. (**A**) Total ion chromatograms recorded in LC–APCI MS experiments in positive ion mode were used to estimate the lipid content in TP extracts by measuring the total peak areas. A typical chromatogram is shown. The apex of the peak is labeled as (Retention Time; Peak Area). (**B**) The effect of age on the amount of detected lipids. Each point is a separate sample. The data are normalized per one tarsal plate. (**C**) A PCA scores plot illustrates clear differences in Meibomian lipids between mice of different age groups, and their clustering patterns. (**D**) A loadings plot was used to identify lipid markers related to MG maturation (red dots). The numbers at each dot indicate the *m*/*z* values of the corresponding analytes. Free cholesterol, wax esters, cholesteryl esters and triacylglycerols were the major discriminating factors between the samples of different ages. Free Chl and triacylglycerols were the dominant features in P0, P3 and P7 samples, while extremely long-chain wax and cholesteryl esters prevailed in older samples.

Initially, the total lipid content of P0 to P30 samples was estimated by integrating total ion chromatograms (TICs) recorded in LC–MS experiments (Figure 1A), as described in our earlier papers [1,2,18]. Both electrospray ionization (ESI) and atmospheric pressure chemical ionization (APCI) approaches were used. The LC–APCI MS TIC peak areas were normalized against the number of tarsal plates (TPs) used to prepare each sample, which allowed for comparison of the total lipid production per TP in mice of different ages. Though not truly quantitative (as it would require the use of an authentic lipid standard for each of the hundreds of lipid components of meibum, resulting in hundreds of calibration curves), this approach clearly visualized the fold changes in meibum production in P0 to P30 samples (Figure 1B).

Then, the TP samples were subjected to unbiased lipidomic LC–MS analyses using the Principal Component Analysis (PCA) approach. The experiments demonstrated a clear inter-group separation of P0 to P30 samples and their intra-group clustering (Figure 1C). The MG lipidomes changed significantly from P0 to P12, after which time point the changes became more gradual, producing visibly overlapping groups of P17, P21, and P30 samples. The loadings plot identified free Chl, CEs, WEs, and TAGs as major discriminating factors that differentiated the samples. For example, high levels of free Chl and TAGs (along with their diacylglycerol fragments, generated spontaneously in the ion source of the mass spectrometer) were characteristic features of P0 to P7 mice, while WEs and CEs were typical of more mature TPs (Figure 1D). Note that the tightness of clustering may have been somewhat affected at earlier stages of MG development due to, at least, two factors: (1) the swiftness of metabolic changes in MGs at the beginning of organogenesis, and (2) the uncertainty in the actual time of birth of the mouse pups, when even a half-day difference may result in detectable changes in their TP lipidomes.

#### 2.1.2. Observation Mass Spectra of Meibomian Lipids

**Figure 2:**
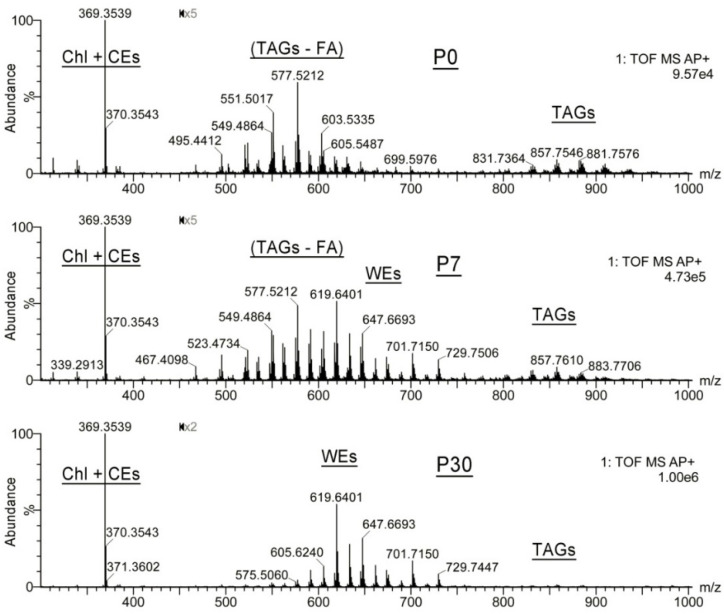
Observation APCI MS PIM spectra of the mouse P0, P7, and P30 Meibomian lipid samples demonstrate a complete lack of typical extremely long-chain Meibomian lipids at P0, and their gradual accumulation in the tarsal plates of maturing mice. The P0 samples were highly enriched with triacylglycerols (detected as intact species and products of their spontaneous in-source fragmentation), free cholesterol, and minor amounts of cholesteryl esters. The P30 sample produced a typical mass spectrum of adult meibum enriched with extremely long-chain wax and cholesteryl esters. The P7 sample assumes an intermediate position between P0 and P30 samples. Note that the regions of the graphs between *m*/*z* 450 and *m*/*z* 1000 were magnified 2 to 5 times for clarity.

The untargeted LC–MS experiments were followed by more focused analyses of mass spectra, which revealed extensive time-dependent changes in specific lipid families of the MG of P0 to P30 mice, illustrated by the observation APCI spectra of three representative samples collected at P0, P7, and P30 (Figure 2). Note that the regions of the graphs between *m*/*z* 450 and *m*/*z* 1000 were magnified 2 to 5 times for better visibility of the MS signals. The experiments revealed that P0 samples had no noticeable pools of typical nonpolar Meibomian lipids, such as ELC WEs and CEs, but were dominated by Chl and shorter-chain CEs (detected using a common analytical fragment *m*/*z* 369.3539 and specific signals of their proton adducts), TAGs (with *m*/*z* values of their proton adducts in the 800 to 1000 range), and their (TAG − FA + H)^+^ fragments formed in-source due to the spontaneous fragmentation of rather unstable (TAG + H)^+^ adducts. By the age of P7, the MG lipidomes already demonstrated detectable accumulation of typical Meibomian-type ELC WEs, such as compounds with an *m*/*z* value of 619.6401, 647.6693, 701.7150, and 729.7506, while at the age of P21 or so the MG lipidome assumed its “adult” composition, and only minor and/or random changes in the balance between major MG lipids were observed. 

**Figure 3:**
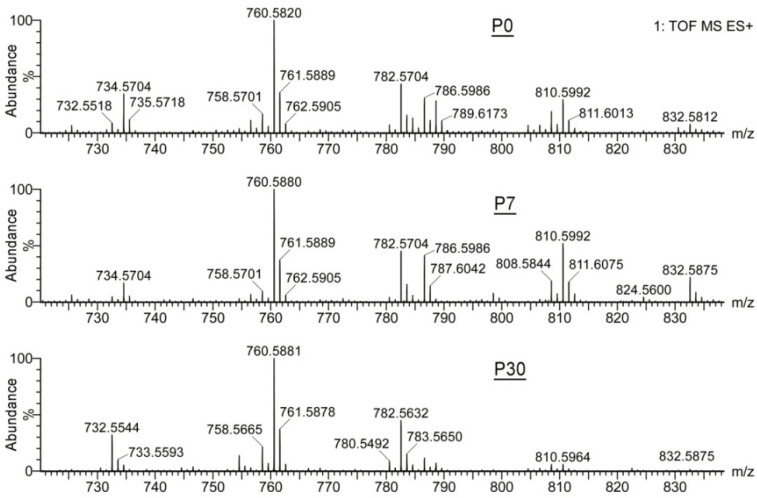
Observation ESI MS PIM mass spectra of samples shown in Figure 2. The sphingomyelin and phospholipid species observed in P0, P7, and P30 samples varied somewhat with the age of the mice, but their intra-group differences were of the same magnitude as the P0 to P30 inter-group differences, and concerned only a few species, such as a phosphatidylcholine with *m*/*z* values of 810.5992 and 832.5875 (a proton and sodium adducts), and its homologue with *m*/*z* 786.5986 and 808.5844 (same adducts). Thus, the differences between the samples may be related to the fact that these ubiquitous phospholipids, being present in all tissues, could originate from the connective tissue that surrounds Meibomian glands and tarsal plates and whose remnants could inadvertently contaminate the samples while dissecting the tissues.

On the other hand, the phosphatidylcholine and sphingomyelin pools of TP Meibomian lipids, analyzed using reverse phase (RP) LC–ESI MS, were more similar to each other, differing only in the ratios of a few homologous members of the families (Figure 3). Notably, phosphatidylcholines C_40_H_80_NO_8_P, C_42_H_82_NO_8_P, C_44_H_84_NO_8_P, and C_44_H_86_NO_8_P (detected as H^+^ and Na^+^ adducts) were invariably the major phospholipid species that were present in all tested samples in similar ratios. 

Thus, it became evident that the most obvious changes in the lipidomes of developing MGs occurred within the families of ELC lipids not found in other tissues and organs, which underwent rapid accumulation after P3 (±1 day) until they reached a steady-state equilibrium at the age of about P21 or so.

#### 2.1.3. Targeted Analysis of Meibomian Lipids

**Figure 4:**
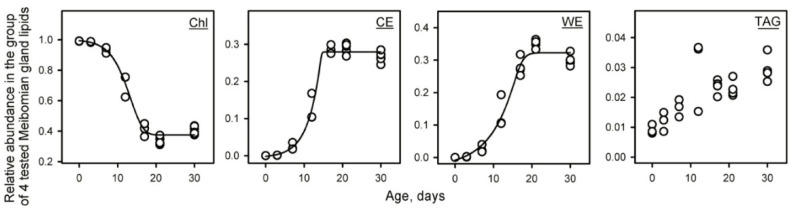
Dynamic changes in the lipid profiles of four typical Meibomian lipids—free cholesterol (Chl), a C_28:1_ FA-based cholesteryl ester (CE), a C_26:0_/C_16:1_ wax ester (WE), and triolein (TAG). The data were normalized so that the total sum of four lipids equals unity. Note a complete absence of the extremely long-chain WE and CE at P0, a noticeable lag in the increases in the CE and WE content during the first few days of maturation, and their exponential growth from P3 to P17. Each data point represents an individual sample from an individual mouse.

Then, a targeted analysis of four typical MG lipids—a free cholesterol (C_27_H_45_, *m*/*z* 369.3521; a proton adduct of the Chl dehydration product), a C_28:1_-CE (C_55_H_102_O_2_N, *m*/*z* 808.7911; an ammonium adduct), a C_26:0_/C_16:1_ WE (Alcohol/Acid; C_42_H_83_O_2_; *m*/*z* 619.6393; a proton adduct), and a TAG triolein (C_57_H_104_O_6_; *m*/*z* 885.7911; a proton adduct)—was conducted (Figure 4). All three latter lipids are major representatives of their respective lipid classes. The ELC WE and CE are expected products of meibogenesis [4] that are not found in food, plasma, or most of the other tissues and organs. Therefore, they were chosen as unique and specific markers, whose dynamics of formation in mouse MGs were to be used to monitor the induction and progression of meibogenesis in maturing mice. Indeed, the CE and WE, which were not present in P0 an P3 samples, increased exponentially between days P3 and P17, while free Chl, which was one of a very few dominant lipids in P0 and P3 samples, quickly declined during the same period. The TAG showed gradual accumulation in the mouse MG lipidome in a rather linear fashion. Note that the sum of *relative* abundances of these four arbitrarily selected markers equals unity, which must not be interpreted as the actual molar ratios of these lipids in meibum; any change in the number of analytes in the test groups will cause changes in the numerical values of their *relative* abundances. However, this approach allowed us to circumvent difficulties in establishing the actual physical sizes of the mouse TPs of different ages, and compensated for the differences in their overall lipid content, as the intra-sample lipid ratios did not depend on either of those factors.

The results of these targeted experiments clearly illustrate the dynamics of changes associated with MG organogenesis and the induction of meibogenesis, and corroborate the results of unbiased untargeted experiments described earlier in the paper. At the ages of P0 to P3, the lipidomes of underdeveloped MGs almost completely lacked typical ELC Meibomian lipids (Figure 2 and Figure 4), and were comprised mostly of Chl and regular TAGs, PLs and SMs. For mice aged to P7 and older, the fraction of true Meibomian lipids emerged and rapidly increased, reaching a plateau between P21 and P30, at which point meibum assumes its normal (or “adult”) composition typical of all previously analyzed specimens from adult WT mice [2,4]. Characteristically, the lipid composition of P0 and P3 mouse TPs closely resembles that of mouse plasma (not shown), which is dominated by regular TAGs, PLs, SMs, and CEs, with a typical FA length of C_16_ to C_20_ or so.

**Figure 5:**
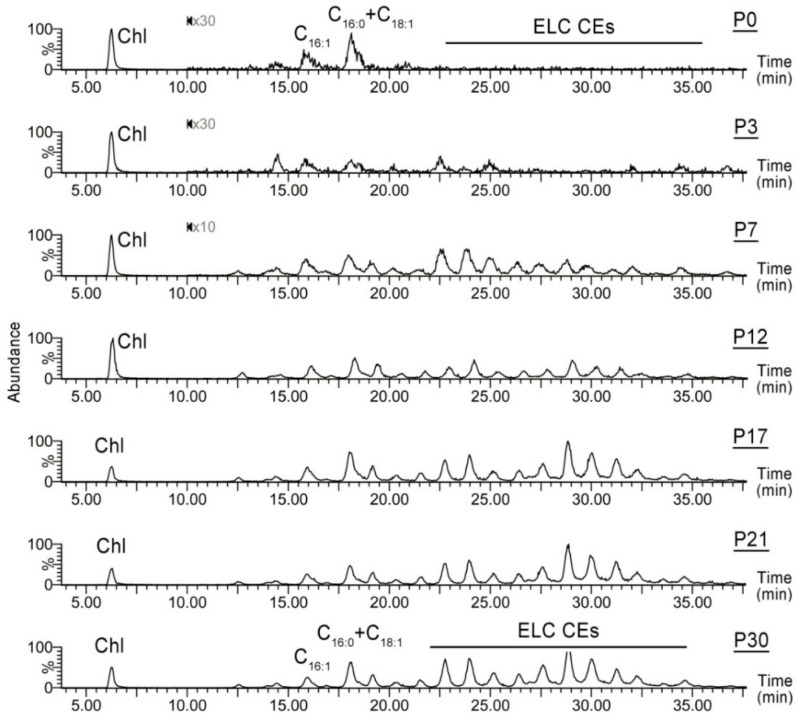
Age-dependent changes in the cholesteryl ester (CE) profiles of mouse Meibomian lipids. The esters were detected using their common analytical fragment with *m*/*z* 369.3539 (see Figure 1). Note the dominance of free cholesterol (Chl) and shorter-chain C_16_ and C_18_-CEs in the P0 samples, and the gradual accumulation of extremely long-chain CEs in the samples obtained from maturing mice. Portions of chromatograms P0, P3, and P7 past 10 min are magnified 10 to 30 times for clarity.

**Figure 6:**
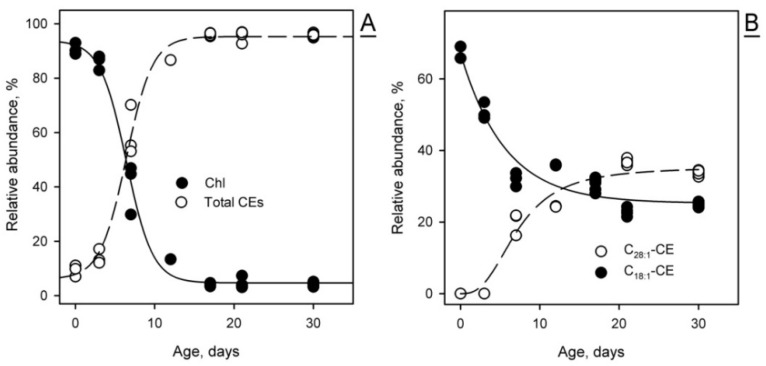
The dynamic changes in free cholesterol (Chl) and cholesteryl esters (CEs) in maturing Meibomian glands of mice. (**A**) The relative abundance of Chl is inversely proportional to the total CE content, and decreases with age. (**B**) The Meibomian CE profile changes with MG maturation: the abundance of shorter chain CEs decreases, while the abundance of extremely long-chain CEs increases. Two specific CEs are shown as examples. Note a lag in the increase in C_28:1_-CE from P0 to P3.

The balance between free Chl and CEs of different types is another parameter that illustrates how mouse maturation affects meibogenesis. Specifically, the CE fraction of P0 and P3 mouse MG lipidomes is dominated by free Chl and normal-length C_14_- to C_20_-CEs, with no detectable ELC CEs (Figure 5). The most common FA residues in those CEs were C_16:0_, C_18:1_, and C_18:2_ FAs. Thus, the pools of total CEs and free Chl demonstrated opposite trends in developing MGs: the molar fraction of Chl rapidly declined, while the pool of CEs rapidly increased (Figure 6A). With maturation, the CE profile progresses toward longer-chain FA (Figure 6B), gradually assuming its typical composition, which was described before for adult mice [2,4]. Note that CEs (unlike WEs) were detectable even at P0, but their FA residues were much shorter than the FA residues of typical Meibomian-type CEs found in mature MGs.

The TAG fraction also increased with maturing from P0 to P30. However, TAGs were already among the major TP lipids of P0 mice (Figure 2). Therefore, their increase was more incremental and less abrupt than the massive changes in CEs, WEs, and other types of true Meibomian lipids (Figure 4).

**Figure 7:**
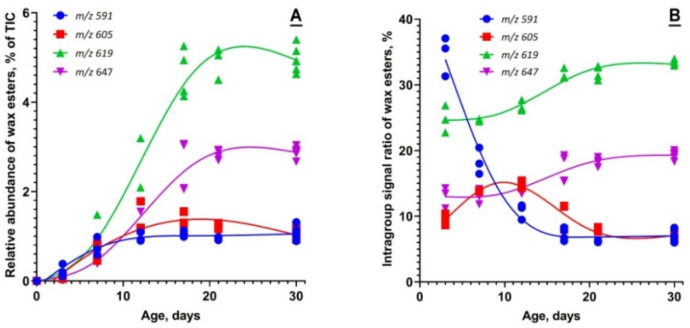
The wax ester (WE) profile of developing and maturing Meibomian glands undergoes age-related changes. (**A**) The relative abundances of four typical WEs with *m*/*z* values of 591.6076, 605.6240, 619.6401, and 647.6693 illustrate the tendency of the overall WE pool to increase from P0 to P30. (**B**) However, the relative abundances of shorter-chain WEs tend to decline, while the relative abundances of extremely long-chain WEs tend to increase.

The pool of WEs also underwent rapid time-dependent transformations (Figure 7). The dynamics of the WE biosynthesis clearly demonstrated the effect of MG maturation on the elongation patterns of WEs. No WEs were detected in P0 samples above the noise level. However, shorter-chain WE, which dominated the WE pool of P3 samples (such as C_40_H_78_O_2_ and shorter), quickly declined by P21 or so. Conversely, the longer-chain WEs, such as C_44_H_86_O_2_, showed an opposite trend: the longest ELC WEs increased in maturing MGs and reached steady-state levels only at about P21 or above.

Thus, LC–MS experiments demonstrated noticeable differences between corresponding sub-pathways of meibogenesis in terms of their initiation and progression at the earlier stages of MG organogenesis.

### 2.2. mRNA Microarray Analyses of Mouse Tarsal Plate Transcriptomes

The next step was to characterize changes in the transcriptomes of developing MG, to determine if there is any correlation between the gene expression patterns (GEPs) and MG lipidomes. As changes in the MG lipidome are expected to be related to the expression levels of various enzymes in mouse TPs, we conducted analyses of their transcriptomes using mRNA microarrays.

#### 2.2.1. Untargeted Transcriptomic Analyses

**Figure 8:**
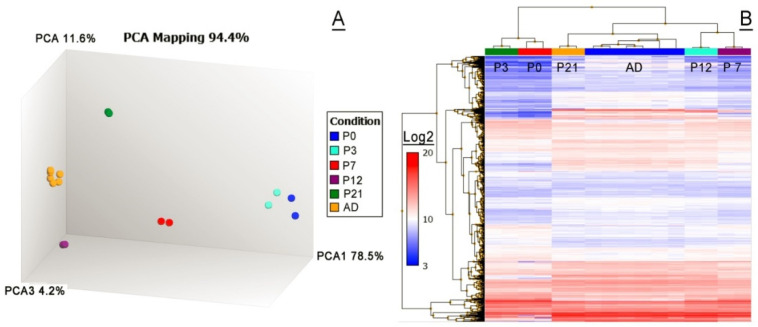
An unbiased transcriptomic analysis of mouse tarsal plates (TPs) using the Principal Component Analysis approach. (**A**) A PCA map of the 1000 most highly expressed genes analyzed using mRNA microarrays. Note a very tight grouping of the samples older than P3. The P0 and P3 samples show a slightly looser grouping due to faster changes in their transcriptomes and the possible impact of a 12 to 24 h uncertainty in the actual time of birth of the mouse pups. (**B**) A hierarchical clustering diagram and a heat map of about 5000 highly expressed genes (on a Log2 scale).

A set of samples ranging from P0 to the adult age of P80 or so (the AD group of samples) was evaluated. The unbiased unsupervised PCA analysis of the samples revealed their tight clustering on the basis of their respective ages, and vast intergroup differences in their transcriptomes. The heatmap and the hierarchical clustering diagrams were used to visualize the similarities and the differences between the samples, and identify clusters of genes that differed significantly between the groups (Figure 8). The 3D-PCA map (Figure 8A) was generated using all detected transcripts (~16,000), while the 5000 most highly expressed transcripts (a limitation imposed by the design of the software) were plotted as the heat map using the Transcriptome Analysis Console (v.4.0.2 from ThermoFisher Scientific; Waltham, MA, USA) (Figure 8B). Note that only the raw data on GEPs that satisfied specific criteria—a *p*-value of <0.05 and an FDR value of <0.05—are illustrated. In addition, noncoding transcripts were excluded from the analysis.

The analyses revealed that large portions of transcriptomes were rapidly and systematically changing in the mouse TPs upon MG organogenesis and maturation. For example, when P3 and AD samples were compared, 7357 transcripts passed the applied filters, of which 3767 genes were found to be upregulated in the adult samples, while 3590 transcripts were downregulated. To identify pathways that are over-represented in the matured mouse MGs in comparison with the developing MGs of P3 mice, the data were analyzed using the Impala Web-based pathway over-representation analysis tool (http://impala.molgen.mpg.de; accessed on 1 July 2022). The pathway information sources included EHMN (The Edinburgh Human Metabolic Network), KEGG (the Kyoto Encyclopedia of Genes and Genomes), Reactome, and Wikipathways databases. The number of overlapping genes, as well as the *p*-values and ***q*** values for genes, were calculated. Due to a more robust statistical nature, with a lower false positive discovery rate, the ***q*** values [22] were used to analyze the data. The list of ~2900 found pathways was filtered to include only those with ***q*** values of ≤0.05, which produced a list of about 70 potentially significant, over-represented pathways. The twelve most significant pathways with gene ***q*** values of ≤0.0001 included the following categories: “Keratinization” (47 overlapping genes of 129 known genes; 47/129), “Formation of cornified envelope” (27/75), Metabolism (176/1952), “Neutrophil degranulation” (65/486), “Metabolism of lipids” (75/645), “Fatty acid metabolism” (33/176), “Oxidative phosphorylation” (26/133), “Innate immune system” (100/1064), “Nuclear receptors meta-pathway” (43/317), “Vitamin D receptor pathway” (30/184), “Developmental biology” (69/676), and “Nonalcoholic fatty liver disease” (26/155).

Next, a list of ~1300 genes that were upregulated in AD samples with an AD/P3 Log2 fold change of ≥2 was analyzed using the Impala’s Wilcoxon pathway enrichment analysis tool. This step produced similar results to the over-representation test, adding “Signal transduction” (124/2432), “Transport of small molecules” (47/641), “Cellular responses to external stimuli” (35/569), and “Cellular response to stress” (34/554) to the list of top upregulated pathways. The full analysis of these changes goes far beyond the scope and size of this paper, is underway, and is to be reported separately.

#### 2.2.2. Transcriptomic Analysis of the Genes of Meibogenesis

**Figure 9:**
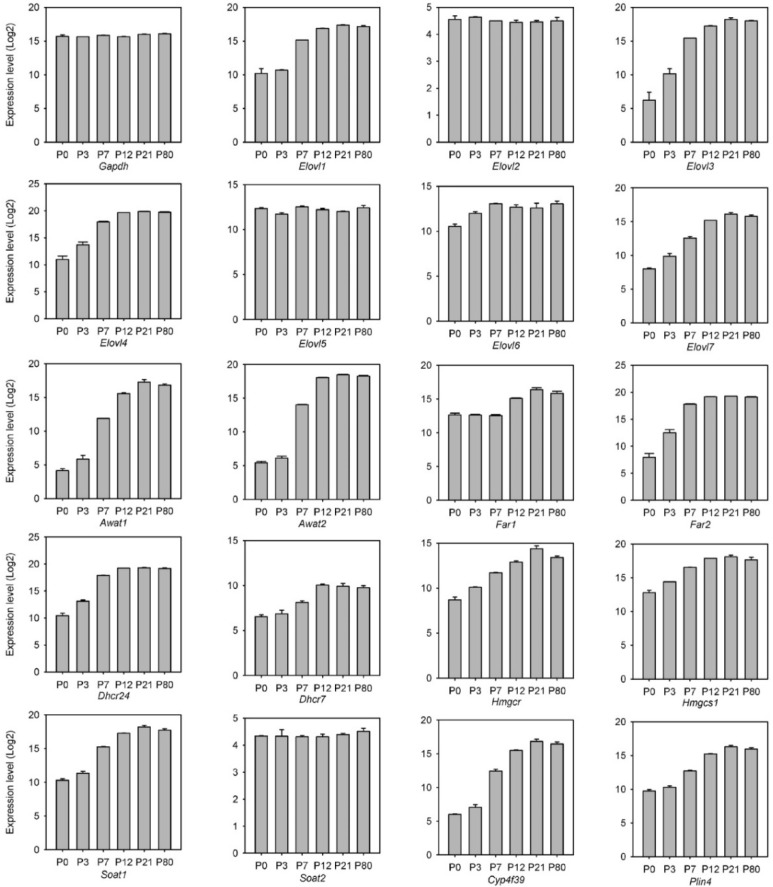
Dynamic changes in the expression levels of selected major genes of meibogenesis in P0 (newborn) pups to P80 (adult) mice measured using mRNA microarrays.

The GEPs in MGs of young adult mice have been partially characterized before, and many major genes that are linked to meibogenesis have been identified [4,6,23]. In the current study, those genes were analyzed against the groups of genes identified in the untargeted transcriptomic experiments described above, including standard house-keeping and reference genes that served as internal standards. The trends in some of these genes are illustrated in Figure 9. The genes included a house-keeping gene *Gapdh*, whose relative levels of expression did not change from P0 to adulthood, and several meibogenesis-related genes shown in the rest of the panels. Other house-keeping genes (e.g., those that encode ribosomal proteins of the S family, *Emc7*, *Hprt*, and others) were evaluated as internal references as well, but did not provide any advantages over *Gapdh.* Due to the very distinctive chemical nature of meibum, whose biosynthesis requires a unique combination of lipid-metabolism related enzymes that is not duplicated in other tissues [4], the functional enrichment analysis of the MG transcriptome using available tools proved to have limited informativity, while the choice of meibogenesis-related genes was based on recent advances in studying meibogenesis [4,6] using various animal models, such as *Elovl1^−/−-^* [7], *Elovl3^−/−^* [9,12], *Elovl4^+/−^* [8], *Elovl 6^−/−-^* [17], *Awat1^−/−^* and *Awat2^−/−^* [13,14,24,25], *Far1^−/−^* and *Far2^−/−^* [15,26], *Soat1^−/−^* [27], *Cyp4f39^−/−^* [16] gene knockout mice, the human MG epithelial cell line (evaluated for the expression of *Hmgcr* [28]), and *Elovl7*-transfected cell lines [11].

Importantly, the expression levels of many of the evaluated genes correlated positively with the overall production of meibum by MGs (Figure 1 and Figure 9). An explosive increase in Meibomian-type ELC WEs and CEs from P0 to P21 coincided with an equally fast accumulation of mRNA transcripts that encode key enzymes of the fatty acid (FA) elongation cycle (e.g., *Elovl1*, *Elovl3*, *Elovl4*, *Elovl6*, and *Elovl7*, but not *Elovl2* and *Elovl5*, which did not change), their reduction into fatty alcohols (*Far1* and *Far2*), esterification into WEs (*Awat1* and *Awat2*) and CEs (*Soat1*, but not *Soat2*), Chl biosynthesis (*Dhcr7*, *Dhcr24*, *Hmgcr*, and *Hmgcs1*), the ω-oxidation of ELC FA (*Cyp4f39*), and lipid storage (*Plin2* and *Plin4*), among other target genes. Note that the data in Figure 9 are presented as Log(2) values, so the actual fold change (FC) in the gene expression levels of, e.g., *Elovl3* from P0 to P21, approaches 4 × 10^3^. The dynamics of biosynthesis of major classes of Meibomian lipids and their individual species showed noticeable lagging during the first days of postnatal development (Figure 4, Figure 5, Figure 6 and Figure 7), but, in general, followed the trajectory of other related genes.

**Figure 10:**
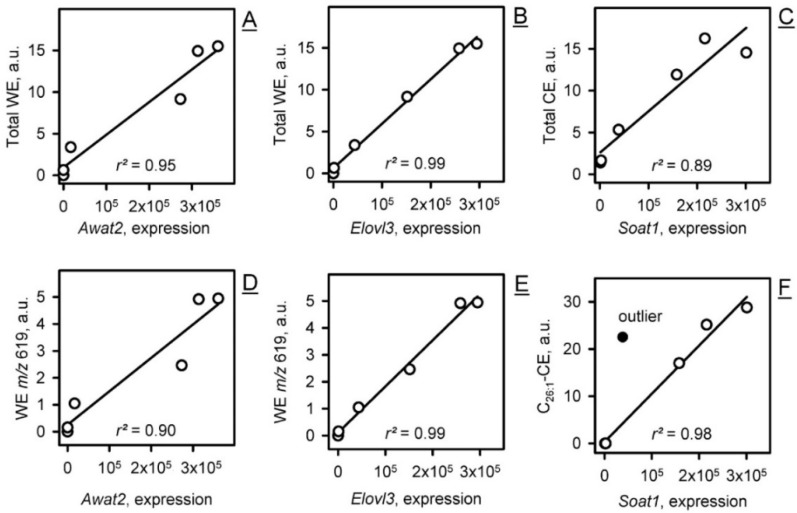
The levels of specific lipid classes and individual lipids in Meibomian lipidomes of P0 to AD mice positively correlated with the levels of expression of related genes. (**A**) Total WEs vs. *Awat2*. (**B**) Total WEs vs. *Elovl3*. (**C**) Total CEs vs. *Soat1*. (**D**) An individual WE with *m*/*z* 619.6393 vs. *Awat2*. (**E**) The same WE vs. *Elovl3*. (**F**) An individual C_28:1_-CE vs. *Soat1*. An outlier is shown as a black dot.

Of practical interest was an observation of a quasi-linear relationship between the levels of expression of many genes and the levels of their respective lipid products in meibum, e.g., between the *Elovl3*/*Awat2* pair and total and individual WEs, between *Soat1* and total and individual CEs, and in other similar cases (Figure 10).

### 2.3. Single-Cell mRNA Analyses of Mouse MGs

**Figure 11:**
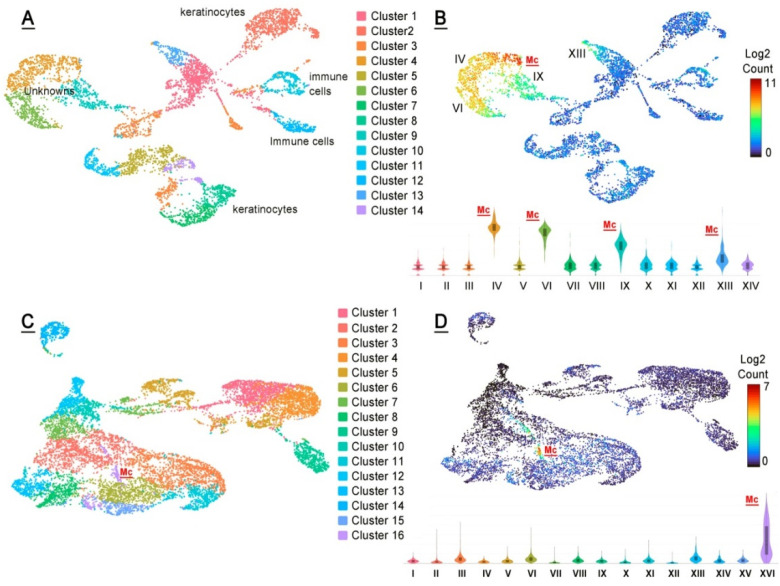
scRNA-seq analyses of mouse tarsal plates. (**A**) Experiments led to the detection of 14 well-defined UMAP cell clusters in AD and P7 mouse tarsal plate samples. (**B**) The genes shown in Table 1 were used as a list of meibogenesis-related features, which resulted in the identification of four prominent cell clusters with high expression levels of those genes, specifically clusters IX, VI, IX, and XIII. Cells that were tentatively identified as meibocytes are labeled in red as “Mc”. (**C**) A UMAP landscape of a P7 sample. Note a very small cluster, XVI, that was linked to meibocytes. (**D**) A UMAP and a violin plot of genes from Table 1 illustrates the genes of meibogenesis are highly expressed only in cell cluster XVI.

Finally, the tarsal plates of AD and P7 mice were subjected to single-cell RNA sequencing (scRNA-seq) (Figure 11). About 6000 cells were analyzed, with ~44,000 mean reads per cell. The total number of detected transcripts was 19,629. The adult AD sample produced 14 distinctively different cell clusters **I** to **XIV**, of which 4 clusters, specifically clusters **IV**, **VI**, **IX** and **XIII**, had high expression values of genes implicated in meibogenesis, and were tentatively assigned to the broadly defined group of meibocytes. The genes with the highest levels of expression included those of the FA elongation cycle (such as *Elovl1*, *Elovl3*, and *Elovl4*), FA reductases (*Far1* and *Far2*), FA desaturases (*Scd1*, *Scd3*, and *Scd4*), FA esterases that produce WEs and CEs (*Awat1*, *Awat2*, and *Soat1*), genes of Chl biosynthesis and metabolism (*Dhcr24*, *Hmgcs*, *Msmo*, and *Hsd17b2*), and a range of other genes that are related to lipid metabolism and storage (Table 1). 

However, the clusters differed in the relative levels of expression of these genes, with cluster **IV** producing the highest levels of the largest number of genes from the list, while cluster **IX**—the lowest, but still more than other 10 clusters identified in the mouse TPs. Due to the high abundance of the genes of meibogenesis, cell clusters **IV**, **VI**, **IX**, and **XIII** were deemed to be related to meibocytes at different states of differentiation/maturation, and/or their progenitor cells. The cell clusters with the highest enrichment levels of these genes were labeled as Mc and highlighted in red font in Figure 11. Using specific marker genes, other cell clusters were tentatively identified as keratinocytes, fibroblasts, epithelial cells, and various immune cells. Importantly, no measurable expression of sebocyte-specific genes *Saa1*/*2* [29] was detected in any of the cell clusters.

Then, the genes listed in Table 1 were analyzed against the P7 dataset, and the clusters of cells with the same set of genes were identified. Of 16 cell clusters, the most highly enriched with meibogenesis-related genes was cluster 16 (also labeled as Mc in the graph). Note a substantially lower number of cells in cluster 16 of the P7 sample compared with cluster 4 of the AD sample, which is consistent with a much lower ratio of Meibomian lipids to other lipids in the TP samples of adolescent mice (Figure 5, Figure 6 and Figure 7). 

**Figure 12:**
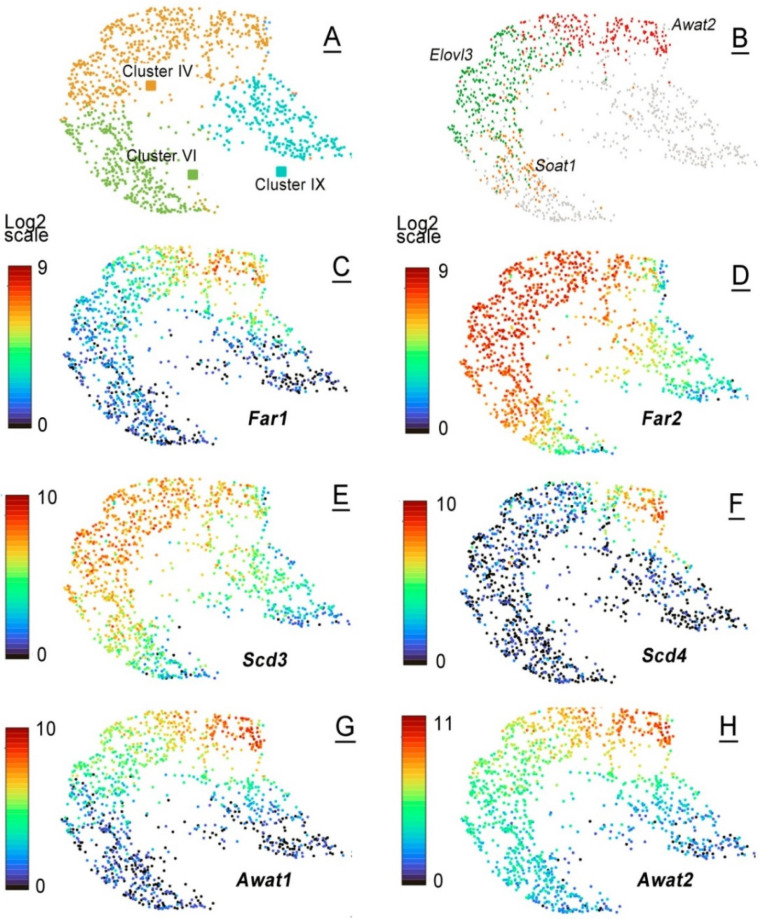
Differential expression of meibogenesis-related genes in clusters and subclusters of AD cells analyzed using the scRNA-seq approach. (**A**) Three major UMAP cell clusters IV, VI, and IX with high expression levels of genes from Table 1 are shown. (**B**) The subclusters of cells with highest expression levels of *Elovl3*, *Awat2*, and *Soat1* imply the existence of unique subpopulations of cells within the three major clusters. (**C**), (**D**), (**E**), (**F**), (**G**), and (**H**) Uneven expression of *Far1*/*Far2*, *Scd3/Scd4*, but not *Awat1/Awat2,* in clusters IV, VI, and IX may lead to differential accumulation of lipids of different types in those cells.

Finally, an observation was made with regard to the different dynamics of changes in the GEPs of individual genes even within individual cell clusters. For example, *Far1* and *Far2*, which are responsible for producing shorter-chain and ELC Meibomian-type fatty alcohols, correspondingly [15,30], are differentially expressed in different cells within the same cell clusters **IV**–**VI** (Figure 12). A similar observation was made for the pairs of genes *Scd3* and *Scd4*, while genes *Awat1* and *Awat2* were expressed in seemingly the same pools of cells in clusters **IV** and **IX**, but differentially in cluster **VI**. A detailed scRNA-seq analysis of the mouse TPs goes beyond the scope of this manuscript and should be conducted in future studies.

**Table 1:** Gene expression patterns in cell clusters of the AD and P7 mouse tarsi. The list of genes is based on the previously published data on the gene expression patterns in mouse and human Meibomian glands. The data are shown as Median Normalized Averages (MNAs) of genes in the clusters calculated in Loupe Browser as the means of observed UMI counts normalized by the size factor for each cell in the respective clusters.

Gene	Cluster IV (AD), MNA	Cluster VI (AD), MNA	Cluster IX (AD), MNA	Cluster XIII (AD), MNA	Cluster XVI (P7), MNA
*Scd1*	217.4	197.7	197.7	107.6	34.0
*Elovl1*	100.4	41.2	40.1	57.9	2.8
*Elovl4*	52.2	19.0	16.1	44.4	3.1
*Far2*	38.7	23.1	18.3	16.7	5.8
*Msmo1*	37.9	35.2	24.3	19.4	14.3
*Awat2*	33.3	1.9	5.0	53.2	0.8
*Dhcr24*	31.1	77.5	73.5	12.6	15.6
*Scd3*	30.6	12.6	14.8	15.3	3.5
*Elovl3*	30.5	16.4	13.0	25.3	8.5
*Soat1*	25.2	22.3	21.6	8.1	10.4
*Hacd2*	23.7	17.1	10.0	16.2	4.2
*Plin2*	20.9	9.5	10.6	35.7	4.3
*Awat1*	18.3	0.3	2.6	18.2	0.6
*Acot1*	16.8	6.3	3.8	13.1	3.0
*Sdr16c5*	15.3	11.3	8.5	10.3	4.0
*Dgat2*	14.7	15.3	9.0	6.6	2.7
*Hacl1*	13.1	6.3	5.2	10.5	2.9
*Hmgcs1*	10.4	27.6	23.1	6.6	15.2
*Dhrs7*	10.4	8.0	5.6	8.2	8.0
*Scd4*	6.6	0.1	1.8	15.8	<0.01
*Far1*	6.4	0.3	2.8	4.7	0.2
*Phyh*	5.9	1.7	1.2	4.3	0.2
*Hsd17b2*	5.2	0.6	0.9	4.4	0.8
*Acadm*	5.2	5.0	2.8	2.7	1.7
*Acaa1b*	5.0	0.5	1.4	8.1	0.6
*Hsd3b6*	3.7	0.4	0.8	2.1	0.6
*Fasn*	3.1	8.2	8.5	1.2	3.7

## 3. Discussion

The data described above clearly illustrate the timeline of changes in the profiles of Meibomian lipids and the amount of meibum produced by MGs, which occur while the mice mature from P0 through adolescence to adulthood. It was reported that MGs become fully developed by the age of P14–P15 [21]. Per our data, it is not until P21 or older that the Meibomian lipidome assumes its final adult composition, and meibogenesis reaches its full capacity (Figure 1, Figure 4, Figure 5, Figure 6 and Figure 7). One of the examples is the Chl, CE, and TAG content, which peaks at P21 or later (Figure 4). Another example is the age-dependent alterations in the elongation patterns (i.e., the balance between shorter- and longer-chain species) of Meibomian CEs and WEs, with longer-chain lipids lagging behind their shorter-chain counterparts (Figure 5, Figure 6 and Figure 7). Therefore, the *anatomical* maturation of the glands does not necessarily mean their *physiological* maturation, as some lipids and lipid classes start to accumulate in the MG lipidome sooner than the others do. As the eyes of mice start to open at P11 or so [https://larc.ucsf.edu/mouse-pup-appearance-age; accessed 1 June 2022], the initial composition of meibum that is produced by MGs of younger pups and delivered onto their ocular surface might be somewhat different from the adult meibum, which may have an effect on the ocular surface’s physiology in general, and the tear film specifically.

The lipid composition of meibum clearly depends on the expression levels of enzymes that are involved in meibogenesis. The pathway over-representation and the Wilcoxon pathway enrichment analyses of P3 and AD transcriptomes identified several pathways that may play significant roles in developing and maturing MGs. They included several pathways expectedly related to developmental biology, keratinization, signal transduction, the transport of small molecules, the biosynthesis of fatty acids, metabolism in general, and lipid metabolism, specifically. However, due to the unique chemical nature of meibum, which is clearly different from typical lipidomes of other cells and tissues, a more focused, evidence-driven approach that was based on the concept of *meibogenesis* [4,6] was chosen.

The mRNA microarray and scRNA-seq analyses of the mouse TPs of different ages provided some insight into the correlations between their GEPs and lipidomes. The timelines of changes in the levels of expression of specific genes allowed for the identification of critically important genes and those that, apparently, did not play a noticeable role in meibogenesis in developing and maturing MGs. As examples, let us consider genes/enzymes related to FA elongation. Of all seven known *Elovls*, the genes that did not undergo any measurable changes were *Elovl2* and *Elovl5*, with *Elovl6* experiencing only an incremental increase from P0 to P7. Considering that ELOVL2 and ELOVL5 act specifically toward *polyunsaturated* FAs (PUFAs), while ELOVL3 and ELOVL1 control the elongation of *saturated* and *monounsaturated* FAs [31,32], the transcriptomic data are consistent with the results of lipidomic experiments, which demonstrated that PUFAs are only minor components of mouse and human meibum [1,2,33], and might not be as relevant to the ocular surface physiology as more saturated FAs.

Similarly, the expression levels of *Soat2* remained steady from P0 to P80, while *Soat1* underwent a rapid increase from P0 to P21, and so did genes that encode enzymes of the Chl biosynthesis pathways *Dhcr7*, *Dhcr24*, *Hmgcr*, and *Hmgcs1* (Figure 9). Their concerted increase, together with *Elovls* and *Soat1*, provided necessary precursors for the biosynthesis of ELC CEs. Note that some of these genes were expressed at relatively high levels in TPs even at P0 and P3, which may partially explain the presence of shorter-chain C_14_–C_18_ CEs in those specimens (Figure 5 and Figure 6), and whose Chl and FA moieties could have been synthesized in situ, or originated from blood lipids [34].

Indicatively, the levels of expression of the major genes of meibogenesis correlated positively with the lipids that are produced by corresponding enzymes (Figure 10). Thus, one may argue that the mRNA expression levels can be rough predictors of the levels of related lipid products, if the genes are highly expressed and the proteins/enzymes they encode are rate-limiting or pivotal points in a biosynthetic pathway.

The scRNA-seq experiments with TPs of P7 and P80 mice visualized multiple cell clusters with unique combinations of expressed genes (Figure 11A). Cell clusters **I**–**XIV** were initially characterized using the default built-in algorithms of the Loupe Browser. Some of the cell clusters were identified as keratinocytes and immune cells, while clusters **IV**, **VI**, **IX**, and **XIII** did not match any known cell types. However, when a list of major genes that had been identified in MGs of mice and humans and linked to meibogenesis (partially shown in Table 1) was used to re-analyze the data, it became evident that those genes were highly expressed in clusters **IV**, **VI**, **IX**, and **XIII**, but not in the other clusters (Figure 11B). Thus, a conclusion was made that these clusters are related to meibogenesis and are likely to be formed of meibocytes at different stages of differentiation/maturation, and/or their progenitor cells. Consistent with the lipidomics data, the P7 sample had a very low number of cells with high levels of expression of the genes listed in Table 1, while the P80 sample had a much more substantial population of the cells of interest.

In addition to the major cell clusters of interest in P7 and P80 samples, multiple *subclusters* of cells with unique GEPs within the major clusters were observed (Figure 12). A detailed analysis of these scRNA-seq features goes beyond the scope of this paper. However, our preliminary data led us to a conclusion that each of the major cell clusters **IV**, **VI**, **IX**, and **XIII** (Figure 12A) consisted, in fact, of a range of subclusters with varying ratios of relevant genes of meibogenesis. For example, cluster **IV** unmistakably had at least two subpopulations of cells that differed in the relative ratios of *Elovl3* and *Awat2* (Figure 12B). Similarly, pairs of genes *Far1/Far2* and *Scd3/Scd4* were differentially expressed in cell clusters **IV**, **VI** and **IX**, forming a few well-defined subclusters. Genes *Awat1/Awat2*, on the other hand, were expressed almost identically in clusters **IV** and **VI**, with *Awat2* appearing at somewhat higher levels in cluster **VI** than *Awat1*.

Finally, an often asked, but insufficiently understood, question about resemblance of Meibomian glands and sebaceous glands (SGs) should be discussed. A comparative lipidomic experiment with human meibum and sebum revealed fundamental dissimilarities between these two secretions [35], which included, among other differences, much higher levels of TAGs, squalene and Chl (also reported by Boughton et al. [36]), and much lower levels of WEs and CEs, in sebum (Figure 3 and Figure 7, *ibid.*). Of major importance are differences in the elongation patterns of WEs and CEs (compare, for example, lipidomes of meibum [1] and sebum [37,38]), and some other features unique to either MGs or SGs, the discussion of which goes beyond the scope of this paper. These dissimilarities in Meibomian and sebaceous lipidomes imply noticeable differences in the molecular machineries that produce meibum and sebum. Considering a rather low presence, and a much smaller size, of true SGs in the TPs of mice, their contribution to both lipidomes and transcriptomes of the latter should be minimal. This conclusion is supported by the absence of any appreciable levels of expression of genes *Saa1* and *Saa2* (identified as reliable markers of in vivo activated SGs and sebocytes [29]) in any of the cell clusters shown in Figure 11 and Figure 12. However, a direct comparative analysis of MGs and SGs in future studies should provide definitive answers to all questions related to similarities and differences between sebogenesis and meibogenesis.

## 4. Materials and Methods

### 4.1. Reagents

Lipid standards were purchased from NuChek Prep. Inc. (Elysian, MN, USA), MilliporeSigma (St. Louis, MO, USA) and Avanti Polar Lipids, Inc. (Birmingham, AL, USA). Chromatography- and mass spectroscopy-grade solvents, such as iso-propanol, acetonitrile, chloroform, methanol, and water, as well as acetic acid, formic acid and ammonium formate, were from MilliporeSigma and ThermoScientific (Waltham, MA, USA). Compressed ultra-high purity gasses (helium and nitrogen) were from local suppliers.

### 4.2. Animals

All animal-related procedures were approved by the Institutional Animal Care and Use Committee of the University of Texas Southwestern Medical Center (UTSW) and conducted in accordance with the Association for Research in Vision and Ophthalmology (ARVO) Statement for the Use of Animals in Ophthalmic and Vision Research.

The experimental animals (the C57Bl/6J founder mice) were purchased from the Wakeland Mouse Breeding Core facility at UTSW and the colony was maintained, bred and aged at UTSW Animal Research Center (ARC) under the constant supervision of veterinarians and trained technicians. The animals were on a 12 h light–dark cycle with unlimited access to food and water. The 2016 Teklad global 16% protein rodent chow (Envigo, Indianapolis, IN, USA) was used throughout the experiments. The actual ages of experimental animals selected for lipidomic experiments were as follows: P0 (actual range P0 to P1), P3 (±1 day), P7 (±1 day), P12 (±1 day), P17 (±1 day), P21 (±1 day), and P30 (±1 day). A one-day uncertainty in the ages is used to account for experimental difficulties in determining the exact times of birth, which sometimes occurred on weekends and/or at night time. The animals selected for transcriptomic experiments were P0, P3, P7, P12, P21, and AD (i.e., P80 ± 10 days). As previous experiments produced no evidence of a noticeable role of sex in meibogenesis in mice and humans [1,2] on the level of gene expression profiles and on the level of meibum lipid profiles, male and female samples were treated as equals and pooled when necessary.

### 4.3. Transcriptomic Analyses of Mouse Tissues

Mice were euthanized with a ketamine/xylazine cocktail and the mouse TPs were immediately excised from the eyelids using a Zeiss Stemi 508 dissecting microscope equipped with a Zeiss CL 6000 LED light source (both from Zeiss, White Plains, NY, USA). Four TPs from each mouse were collected and dissected free from other ocular tissues as described recently on several occasions [2,9,12,27]. The specimens were kept chilled during the procedure using ice. The TPs collected for transcriptomic analyses were stored in the RNAlater solution (Qiagen, Germantown, MD, USA) in glass vials until they were analyzed at the UTSW Microarray and Immune Phenotyping Core facility using Clariom D microarrays (Affymetrix, Santa Clara, CA, USA) as described earlier [2]. Only samples with RNA integrity numbers ≥9 were included in the analyses. The RNA expression data were imported into the Expression Console (v.1.4.1.36, Affymetrix), processed and analyzed in the Transcriptome Analysis Console (v.4.0.1, from the same manufacturer). Specific criteria—a linear fold change (LFC) less than −2, or more than +2, and *p* ≤ 0.05—were used to determine the statistical significance of the differences between the samples.

### 4.4. Single Cell RNA Sequencing

The TP specimens collected for the scRNA-seq experiments from 3 adult mice and 7 pups (4 TPs from each mouse) of mixed sexes were analyzed at the UTSW Microbiome Research Laboratory. The TPs were digested in 0.25% collagenase D (MilliporeSigma), 1.5 mg/mL Dispase II (Roche), 0.04% bovine serum albumin (BSA) in Hanks Balanced Salt Solution (ThermoFisher Scientific) for 1 h at 37 °C on a shaker. MGs were further isolated from extraneous tissue under a dissecting microscope and incubated in 0.05% Trypsin-EDTA for 5 min. MGs were gently triturated into a single cell suspension with a 10 mL pipette, washed with 10% fetal bovine serum-phosphate buffered saline and strained with a 70 μm mesh cell strainer. Cells were washed 3 times with 0.04% BSA in PBS without magnesium or calcium to ensure the removal of materials that inhibit reverse transcription. The final cell pellet was suspended in 1 mL 0.04% BSA in phosphate buffered saline (−Mg^2+^, −Ca^2+^) and submitted to the Core facility within 3 to 4 h after euthanasia. The cell suspension was filtered through a 40 μm mesh strainer and cell count and viability were checked using the TC-20 cell counter prior to scRNA-seq analyses.

The results of scRNA-seq experiments were analyzed and visualized using built-in algorithms of the Loupe Browser v.5.1.0 (from 10x Genomics, Pleasanton, CA, USA). Log2 fold changes in the gene expression values were used throughout.

### 4.5. Lipidomic Analyses of Mouse Tarsal Plates

Between two and four excised TPs from each mouse were used for lipid extraction. Though a larger number of TP improved the signal-to-noise ratio and minimized the effects of the background noise, two TPs from an individual animal were sufficient for all lipidomic experiments. Typically, TPs were incubated in ~1 mL of chloroform/methanol = 2/1 (vol/vol) solvent mixture at +4 °C overnight, the solvent was transferred into a glass vial, and the extraction was repeated twice for 15 min each time. No homogenization of the tissues was needed because of the small size of the mouse TP. The extracts were combined, brought to dryness under a stream of compressed nitrogen at 37 °C, and redissolved in 1 mL of iso-propanol. The samples were stored at −80 °C until the analyses.

Liquid chromatography–mass spectrometry analyses were performed as described before [27,34] using a Waters Synapt G2-Si high-resolution quadruple Time-of-Flight (qToF) mass spectrometer (Waters Co., Milford, MA, USA). Two ion sources (an ESI low flow and an APCI IonSabre-II) with a Zspray/LockSpray housing were used. The analytes were detected in both positive and negative ion modes using either sensitivity (with resolution R > 10,000 at full width at half maximum, FWHP) or high resolution (R > 40,000 at FWHP) settings.

Lipid extracts were analyzed using isocratic and gradient reverse phase ultra high-performance liquid chromatography (RP-UPLC) on, respectively, C_8_ and C_18_ BEH Acquity UPLC columns (from Waters Co.). The isocratic elution of the lipid analytes from the C_8_ column (2.1 mm × 100 mm, with 1.7 μm particle size) was conducted using iso-propanol/10 mM aqueous ammonium formate = 95/5 (vol/vol) solvent mixture **1**, while the C_18_ column (1.0 mm × 100 mm, 1.7 μm) was used for gradient RP-UPLC using elution with acetonitrile/iso-propanol/10 mM aqueous ammonium formate solvent mixture **2** as described before [1,2,4]. Note that the ESI experiments were conducted using the C_18_ column exclusively, as solvent mixture **1** did not lead to the formation of a sufficient number of ionized lipid species, while the acetonitrile/iso-propanol/10 mM aqueous ammonium formate solvent mixture **2** did. The APCI experiments, on the other hand, were successfully conducted with either column/eluent combination. Importantly, the latter combination allowed for reliable detection and quantitation of free Chl, which was poorly ionizable/undetectable under the conditions of ESI analyses in our hands, and in the hands of other researchers [39,40,41].

The LC–MS results were analyzed using the MassLynx, MS^E^ Data Viewer, and EleComp programs obtained from Water Corp., Inc., and the Principal Component Analysis approaches as described earlier [27,35].

### 4.6. Data Computation and Statistical Analyses

The experimental results of lipidomic experiments were analyzed using a MassLynx software package v.4.1 and Progenesis QI (from Waters Corp./Nonlinear Dynamics; Milford, MA, USA) and SigmaStat v.3.5 (from Systat Software, Inc., San Jose, CA, USA). The elemental CHO compositions of the lipids were derived using the EleComp feature of the MassLynx. The accuracy of the analyses was better than 3 mDa for the 100 to 2000 Da range, which, in combination with MS/MS analysis and the usage of authentic lipid standards, has made it possible to reliably identify analytes of interest.

## 5. Conclusions

A plausible conclusion that can be derived from the observations presented and discussed above is the simultaneous presence of a wide variety of lipid-producing cells in MG that can, potentially, contribute to the Meibomian transcriptome and lipidome, and whose ratios in maturing MGs change with time. These cells can potentially be either developing/maturing meibocytes, or progenitor cells that end up forming a few (or several) distinctively different cell populations in mouse MGs. In addition, we cannot exclude a possibility of their unique (i.e., specific only to a particular subpopulation of the cells) contributions to meibum, especially in TPs of different ages and in meibocytes at different stages of differentiation and maturation. These hypotheses need to be verified in future experiments, using a combination of methods that are currently being developed.

## Data Availability

All the data that are pertinent to the discussion are included in the paper. Once the study has been completed, the results will be published in separate manuscripts and deposited according to the then-current regulations.
